# Supplementary material for: Novel tau biomarkers phosphorylated at T181, T217 or T231 rise in the initial stages of the preclinical Alzheimer’s continuum when only subtle changes in Aβ pathology are detected
Source: EMBO Mol Med. 2020 Nov 10;12(12):e12921. doi: 10.15252/emmm.202012921 (PMC7721364; doi:10.15252/emmm.202012921)
Supplement: Supplementary file 1 — Appendix [file EMMM-12-e12921-s001.docx]

**Appendix**

**Table of contents**

[**Appendix Table S1. ROC analyses to discriminate cognitively unimpaired Aβ-positive from Aβ-negative individuals** 2](#_Toc50558824)

**Appendix Table S1. ROC analyses to discriminate cognitively unimpaired Aβ-positive from Aβ-negative individuals**

|  | **AUC** (95% CI) **CU Aβ+ *vs* Aβ-** | | |
| --- | --- | --- | --- |
|  | **CSF Aβ42/40**  (A-: 250; A+: 131) | **Aβ PET visual read**  (A-: 287; A+: 42) | **Aβ PET CL12**  (A-: 278; A+: 53) |
| **CSF Mid-p-tau181** | 0.683 (0.627 -0.739) | 0.814 (0.748 – 0-881) | 0.748 (0.673 – 0.824) |
| **CSF N-p-tau181** | 0.763 (0.710 – 0.816)^1^ | 0.858 (0.787 – 0.928)^11^ | 0.855 (0.794 – 0.916)^1^ |
| **CSF N-p-tau217** | 0.794 (0.744 – 0.844)^1^ | 0.883 (0.825 – 0.941)^12^ | 0.875 (0.819 – 0.931)^1^ |
| **CSF Mid-p-tau231** | 0.809 (0.761 – 0.856)^1,2^ | 0.894 (0.833 -0.954)^1^ | 0.894 (0.845 -0.943)^1,19^ |
| **CSF t-tau** | 0.669 (0.612 – 0.726)^3,4,5,6^ | 0.793 (0.725 – 0.862)^6,13,14,15^ | 0.722 (0.646 – 0.799)^1,4,5,6^ |
| **Plasma N-p-tau181** | 0.670 (0.612 – 0.728)^7,8,9^ | 0.671 (0.581 -0.762)^4,5,6,16,17^ | 0.729 (0.656 – 0.801)^6,20,21^ |
| **Plasma NfL** | 0.626 (0.567 – 0.685)^5,6,10^ | 0.660 (0.574 – 0.745)^4,5,6,12,18^ | 0.628 (0.545 – 0.710)^4,5,6,22,23,24^ |

ROC analyses to test whether each p-tau biomarker discriminates between Aβ-positive (A+) and Aβ-negative individuals (A-), as defined by the CSF Aβ42/40 ratio, Aβ PET visual read or Aβ PET using a cutoff of CL12. We also included CSF t-tau and plasma NfL for comparison. Aβ PET was performed in 331 participants and Centiloid scale was available in all of them; two participants did not have visual read assessment.

AUCs differences were tested using the DeLong test. The significant differences were:

^1^*P* < 0.0001 compared to CSF Mid-p-tau181

^2^*P* = 0.005 compared to CSF N-p-tau181

^3^*P* = 0.007 compared to CSF Mid-p-tau181

^4^*P* < 0.0001 compared to CSF N-p-tau181

^5^*P* < 0.0001 compared to CSF N-p-tau217

^6^*P* < 0.0001 compared to CSF Mid-p-tau231

^7^*P* = 0.008 compared to CSF N-p-tau181

^8^*P* = 0.0005 compared to CSF N-p-tau217

^9^*P* = 0.0002 compared to CSF Mid-p-tau231

^10^*P* = 0.0001 compared to CSF N-p-tau181

^11^*P* = 0.015 compared to CSF Mid-p-tau181

^12^*P* = 0.003 compared to CSF Mid-p-tau181

^13^*P* = 0.001 compared to CSF Mid-p-tau181

^14^*P* = 0.001 compared to CSF N-p-tau181

^15^*P* = 0.0004 compared to CSF N-p-tau217

^16^*P* = 0.002 compared to CSF Mid-p-tau181

^17^*P* = 0.008 compared to CSF t-tau

^18^*P* = 0.011 compared to CSF t-tau

^19^*P* = 0.045 compared to CSF N-p-tau181 (non-significant after FDR-correction)

^20^*P* = 0.002 compared to CSF N-p-tau181

^21^*P* = 0.0008 compared to CSF N-p-tau217

^22^*P* = 0.008 compared to CSF Mid-p-tau181

^23^*P* = 0.045 compared to CSF t-tau (non-significant after FDR-correction)

^24^*P* = 0.031 compared to plasma N-p-tau181

The *P*-values shown are not corrected for multiple comparisons. We additionally computed the *P*-values applying a false discovery rate (FDR) multiple comparison correction following the Benjamini-Hochberg procedure and all *P*-values remain significant if not otherwise indicated.

Abbreviations: Aβ42, amyloid-β 42; Aβ40, amyloid-β 40; AUC, area under the curve; CI, confidence interval; CL, Centiloid; CSF, cerebrospinal fluid; Mid, mid-region; N, N-terminal; NfL, neurofilament; p-tau, phosphorylated tau; t-tau, total tau; PET, positron emission tomography.
