# Supplementary material for: Novel tau biomarkers phosphorylated at T181, T217 or T231 rise in the initial stages of the preclinical Alzheimer’s continuum when only subtle changes in Aβ pathology are detected
Source: EMBO Mol Med. 2020 Nov 10;12(12):e12921. doi: 10.15252/emmm.202012921 (PMC7721364; doi:10.15252/emmm.202012921)
Supplement: Supplementary file 2 — Expanded View Figures PDF [file EMMM-12-e12921-s002.pdf]

Expanded View Figures

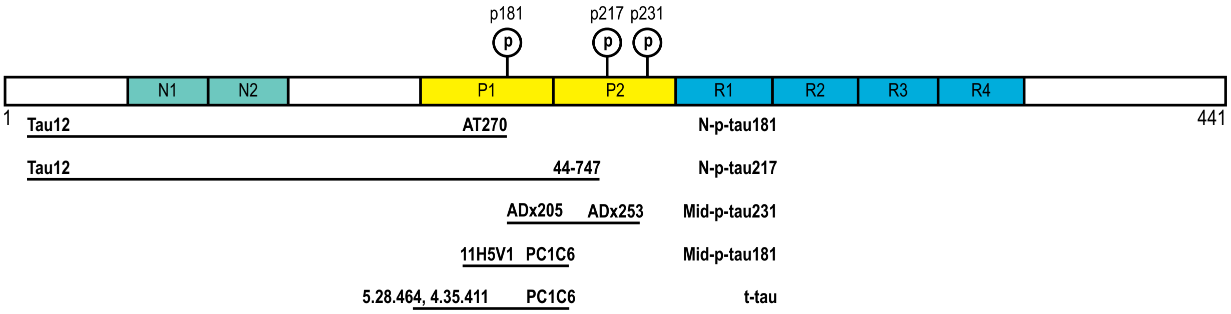

Figure EV1. Diagram of tau and the antibodies used in the assays.

Tau protein domains and the fragments recognized by the combination of antibodies of the assays used in this study. We show the longest form of tau (2N4R), which comprises 441 amino acids. The two N-terminal domains (N) are depicted in green, the two proline-rich domains (P) in yellow and the four microtubule-binding domains (R) in blue. The phosphorylation sites identified by the assays are also shown. Details of the assays can be found in the methods section. Abbreviations: Mid, mid-region; N, N-terminal; p-tau, phosphorylated tau

 © 2020 The Authors

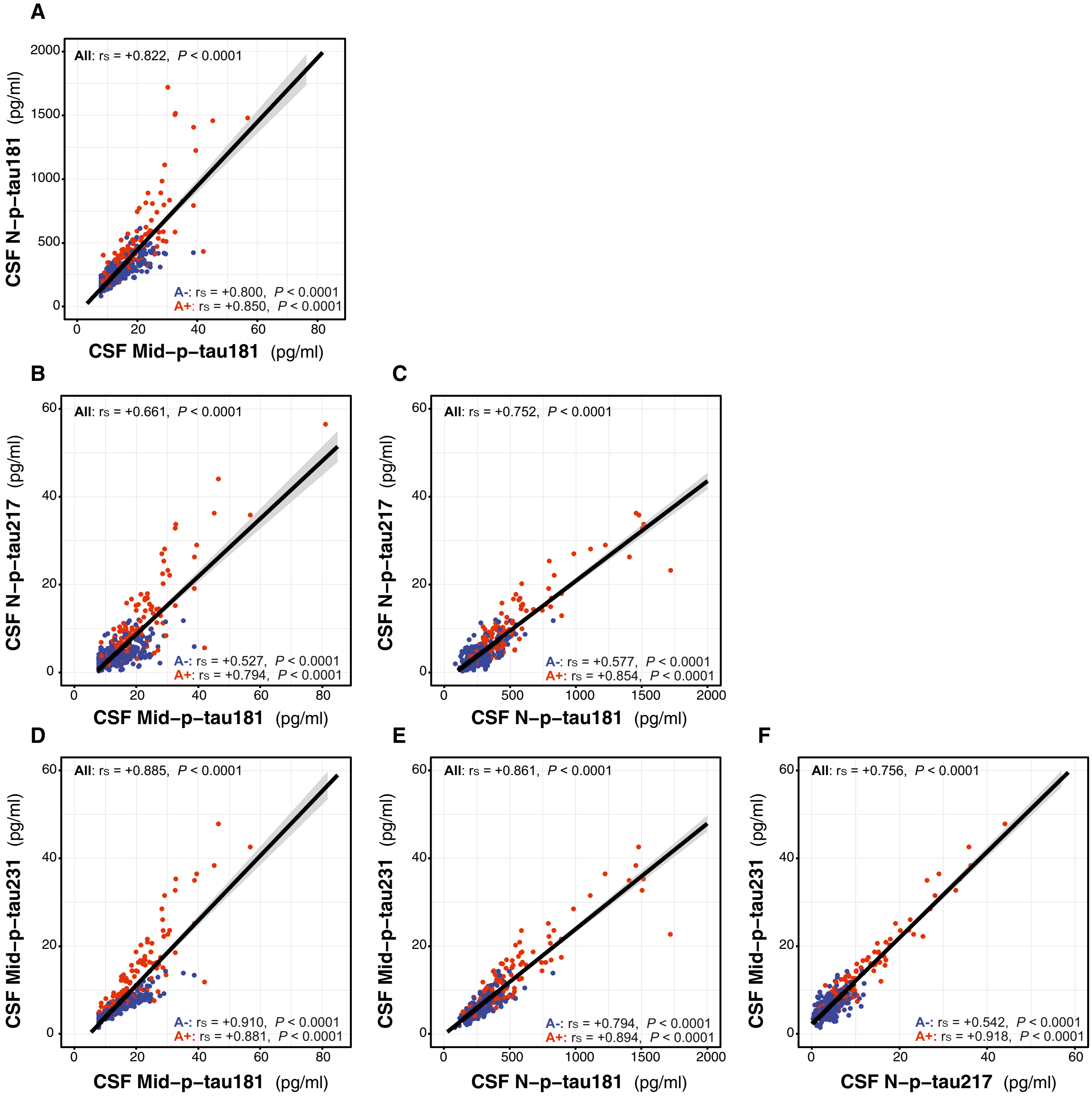

Figure EV2. Correlations between CSF p-tau biomarkers.

A–F Scatter plots depicting the correlations between the CSF p-tau biomarkers. We computed the Spearman's correlation coefficient (r_s) and the *P*-value for the whole cohort (*n* = 381) and for the A− (*n* = 250; blue dots) and A+ (*n* = 131; red dots) groups. The solid lines indicate the regression line and the 95% confidence intervals. Abbreviations: CSF, cerebrospinal fluid; Mid, mid-region; N, N-terminal; p-tau, phosphorylated tau.

© 2020 The Authors

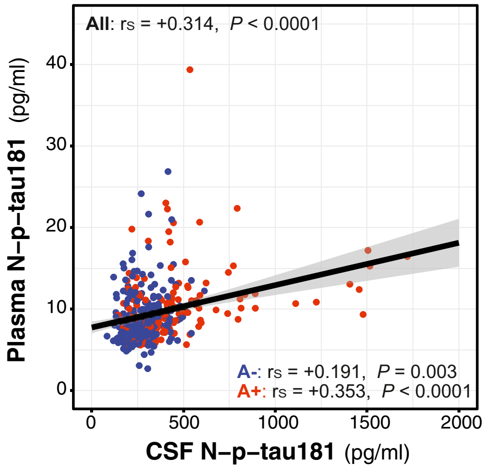

Figure EV3. Correlation between CSF and plasma N-p-tau181.

N-p-tau181 was measured with the same assay in matched CSF and plasma samples. Scatter plots depicting the correlations between the CSF and plasma measurements. We computed the Spearman's correlation coefficient (r_s) and the *P*-value for the whole cohort ($n = 381$) and for the A− ($n = 250$; blue dots) and A+ ($n = 131$; red dots) groups. The solid lines indicate the regression line and the 95% confidence intervals. Abbreviations: CSF, cerebrospinal fluid; N, N-terminal; p-tau, phosphorylated tau.

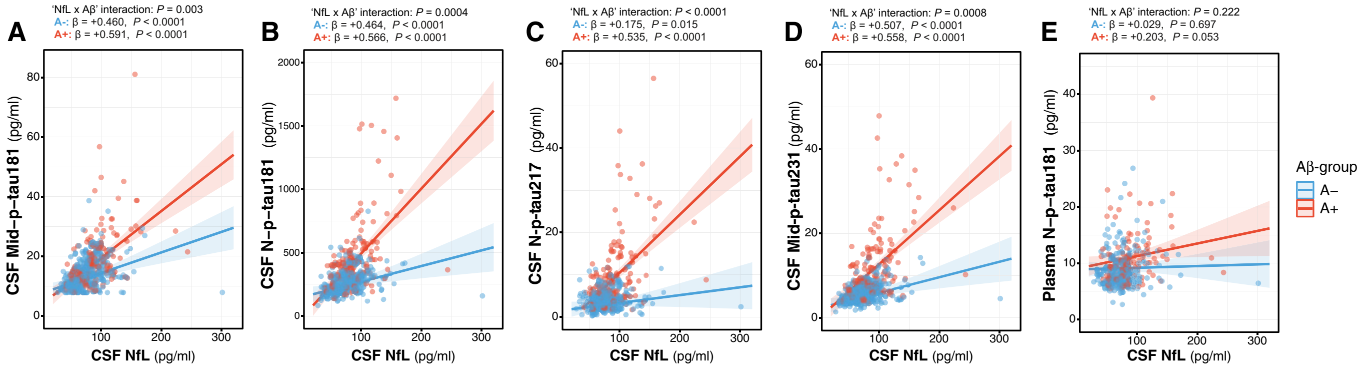

Figure EV4. Associations of p-tau biomarkers and CSF NfL.

A–E Scatter plots showing the association of each of the p-tau biomarkers with CSF Neurofilament light (NfL) in the Aβ-negative (A−; blue) and the Aβ-positive (A+; red) groups. The solid lines indicate the regression line and the 95% confidence intervals for each of the groups. For each group, the standardized regression coefficients (β) and the *P*-values were computed using a linear model adjusting for age and sex. We also computed the "CSF NfL × Aβ status" interaction term. Abbreviations: CSF, cerebrospinal fluid; Mid, mid-region; N, N-terminal; p-tau, phosphorylated tau.

 © 2020 The Authors

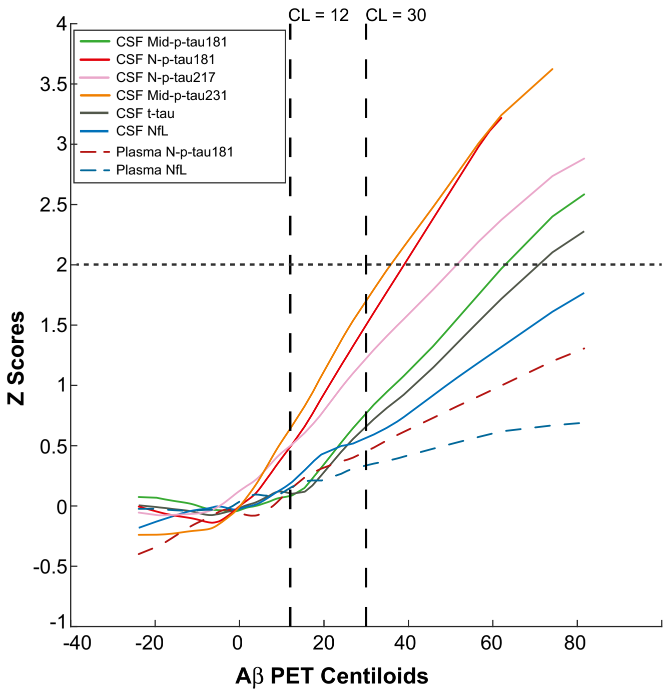

Figure EV5. Trajectories of the p-tau biomarkers as a function of Aβ PET.

The graphs represent the *z*-scores changes of each CSF biomarker as a function of Aβ PET Centiloid scale (instead of CSF Aβ42/40 as proxy of disease progression) using a robust local weighted regression method. The *z*-scores were calculated using the mean and the SD of each CSF biomarker in the A−T− group as a reference. The solid lines depict the trajectory of each CSF biomarker. The dashed lines depict the trajectories of the plasma biomarkers. The vertical black dashed lines indicate the CL = 12 and CL = 30 cut-offs. Consistent with the findings using CSF Aβ42/40 as a proxy of disease progression, the CSF p-tau biomarkers reach the 2 *z*-scores (depicted with an horizontal line) with the following sequence: Mid-p-tau231, N-p-tau181, N-p-tau217, Mid-p-tau181 and T-tau. Abbreviations: CSF, cerebrospinal fluid; Mid, mid-region; NfL, Neurofilament light; N, N-terminal; p-tau, phosphorylated tau; t-tau, total tau.

© 2020 The Authors
