# Supplementary material for: Novel tau biomarkers phosphorylated at T181, T217 or T231 rise in the initial stages of the preclinical Alzheimer’s continuum when only subtle changes in Aβ pathology are detected
Source: EMBO Mol Med. 2020 Nov 10;12(12):e12921. doi: 10.15252/emmm.202012921 (PMC7721364; doi:10.15252/emmm.202012921)
Supplement: Supplementary file 3 — Table EV1 [file EMMM-12-e12921-s003.docx]

**Table EV1. Association of p-tau biomarkers and age in each Aβ status group.**

|  | **Total**  (n = 381) | | **A-**  (n = 250) | | **A+**  (n = 131) | | **‘Age x Aβ status’**  interaction |
| --- | --- | --- | --- | --- | --- | --- | --- |
| **p-tau biomarkers** | **β (SE)** | ***P*** | **β (SE)** | ***P*** | **β (SE)** | ***P*** | ***P*** |
| **CSF Mid-p-tau181** | +0.228 (0.050) | <0.0001* | +0.089 (0.064) | 0.163 | +0.314 (0.083) | 0.0002* | 0.019* |
| **CSF N-p-tau181** | +0.249 (0.051) | <0.0001* | +0.100 (0.064) | 0.121 | +0.311 (0.084) | 0.0003* | 0.009* |
| **CSF N-p-tau217** | +0.230 (0.050) | <0.0001* | +0.053 (0.064) | 0.411 | +0.311 (0.084) | 0.0003* | 0.011* |
| **CSF Mid-p-tau231** | +0.242 (0.050) | <0.0001* | +0.086 (0.064) | 0.179 | +0.294 (0.084) | 0.0007* | 0.010* |
| **Plasma N-p-tau181** | +0.170 (0.051) | 0.001* | +0.122 (0.064) | 0.058 | +0.142 (0.090) | 0.109 | 0.897 |

For each p-tau biomarker we computed the linear regression standardized coefficients (β) and standard errors (SE) in the total sample and after stratifying for Aβ-negative (A-) and Aβ-positive (A+) groups. We also computed the *P*-value for the interaction term ‘Age x Aβ status’. All analyses were adjusted by age and sex. *Significant differences.

Abbreviations: CSF, cerebrospinal fluid; Mid, mid-region; N, N-terminal; p-tau, phosphorylated tau.
