# Supplementary material for: Novel tau biomarkers phosphorylated at T181, T217 or T231 rise in the initial stages of the preclinical Alzheimer’s continuum when only subtle changes in Aβ pathology are detected
Source: EMBO Mol Med. 2020 Nov 10;12(12):e12921. doi: 10.15252/emmm.202012921 (PMC7721364; doi:10.15252/emmm.202012921)
Supplement: Supplementary file 4 — Table EV2 [file EMMM-12-e12921-s004.docx]

**Table EV2. Association of p-tau biomarkers and Aβ PET Centiloid scale stratifying by Aβ status (as defined by Aβ PET CL12 cutoff).**

|  | **Aβ PET CL12** (n = 331) | | | | | | |
| --- | --- | --- | --- | --- | --- | --- | --- |
|  | **A-**  (n = 278) | | | **A+**  (n = 53) | | | **‘Aβ PET**  **x**  **Aβ status’**  interaction |
|  | **β (SE)** | ***P*** | **eta^2^** | **β (SE)** | ***P*** | **eta^2^** | ***P*** |
| **CSF Mid-p-tau181** | +0.058 (0.060) | 0.339 | 0.003 | +0.483 (0.118) | 0.0002* | 0.254 | 0.009* |
| **CSF N-p-tau181** | +0.115 (0.060) | 0.054 | 0.014 | +0.628 (0.132) | <0.0001* | 0.324 | 0.004* |
| **CSF N-p-tau217** | +0.176 (0.060) | 0.003* | 0.031 | +0.610 (0.110) | <0.0001* | 0.391 | 0.246 |
| **CSF Mid-p-tau231** | +0.165 (0.059) | 0.006* | 0.027 | +0.606 (0.119) | <0.001* | 0.350 | 0.040* |
| **Plasma N-p-tau181** | +0.130 (0.060) | 0.032* | 0.017 | +0.253 (0.156) | 0.112 | 0.053 | 0.859 |

For each p-tau biomarker we computed the linear regression standardized coefficients (β) and standard errors (SE) as a function of Aβ PET Centiloid scale. The analyses were performed after stratifying for Aβ-negative (A-) and Aβ-positive (A+) groups, as defined by Aβ PET CL12. We report the eta-squared (eta^2^) as a measure of the effect size. We also computed the *P*-value for the interaction term ‘Aβ biomarker x Aβ status’. All analyses were adjusted by age and sex. *Significant differences.

Abbreviations: CL, Centiloid; CSF, cerebrospinal fluid; Mid, mid-region; N, N-terminal; PET, positron emission tomography; p-tau, phosphorylated tau.
