# Supplementary material for: Novel tau biomarkers phosphorylated at T181, T217 or T231 rise in the initial stages of the preclinical Alzheimer’s continuum when only subtle changes in Aβ pathology are detected
Source: EMBO Mol Med. 2020 Nov 10;12(12):e12921. doi: 10.15252/emmm.202012921 (PMC7721364; doi:10.15252/emmm.202012921)
Supplement: Supplementary file 5 — Table EV3 [file EMMM-12-e12921-s005.docx]

**Table EV3. Concentrations (pg/ml) and between run variations of the novel p-tau assays.**

|  | **IQC 1** | | **IQC2** | | **IQC3** | |
| --- | --- | --- | --- | --- | --- | --- |
|  | **Mean ± SD** | **Between-run CV%** | **Mean ± SD** | **Between-run CV%** | **Mean ± SD** | **Between-run CV%** |
| **CSF N-p-tau181** | 6.6 ± 1.1 | 17.3 | 27.0 ± 1.8 | 6.6 | - | - |
| **CSF N-p-tau217** | 5.6 ± 0.9 | 16.4 | 7.0 ± 0.7 | 9.3 | 9.8 ± 1.1 | 11.5 |
| **CSF Mid-p-tau231** | 4.2 ± 0.1 | 4.3 | 10.9 ± 0.1 | 2.6 | 25.4 ± 0.2 | 3.2 |
| **Plasma N-p-tau181** | 5.3 ± 0.3 | 5.9 | 13.9 ± 0.5 | 3.6 | - | - |

The internal quality control (IQC) samples were EDTA plasma samples that were stored at -80ºC and used once. Each IQC sample was analysed in duplicates at the start and the end of each run.

Abbreviations: CSF, cerebrospinal fluid; Mid, mid-region; N, N-terminal; p-tau, phosphorylated tau.
